# Inshore Ship Detection Based on Level Set Method and Visual Saliency for SAR Images

**DOI:** 10.3390/s18113877

**Published:** 2018-11-11

**Authors:** Tao Xie, Weike Zhang, Linna Yang, Qingping Wang, Jingjian Huang, Naichang Yuan

**Affiliations:** 1 State Key Laboratory of Complex Electromagnetic Environment Effects on Electronics and Information System, National University of Defense Technology, Changsha 410073, China; xietao09@nudt.edu.cn (T.X.); xdwdz2010@163.com (W.Z.); hjjfh2003@aliyun.com (J.H.); yuannaichang@hotmail.com (N.Y.); 2 College of Information and Communication, National University of Defense Technology, Xi’an 710106, China; yanglinna3@163.com

**Keywords:** inshore ship detection, level set method, visual saliency, active contour, computer vision, synthetic aperture radar (SAR)

## Abstract

Inshore ship detection is an important research direction of synthetic aperture radar (SAR) images. Due to the effects of speckle noise, land clutters and low signal-to-noise ratio, it is still challenging to achieve effective detection of inshore ships. To solve these issues, an inshore ship detection method based on the level set method and visual saliency is proposed in this paper. First, the image is fast initialized through down-sampling. Second, saliency map is calculated by improved local contrast measure (ILCM). Third, an improved level set method based on saliency map is proposed. The saliency map has a higher signal-to-noise ratio and the local level set method can effectively segment images with intensity inhomogeneity. In this way, the improved level set method has a better segmentation result. Then, candidate targets are obtained after the adaptive threshold. Finally, discrimination is employed to get the final result of ship targets. The experiments on a number of SAR images demonstrate that the proposed method can detect ship targets with reasonable accuracy and integrity.

## 1. Introduction

Ship detection for synthetic aperture radar (SAR) images is of great importance in both military and commercial applications [1,2,3]. The technology of ship detection for SAR images has made great progress [4,5,6], while fewer works are available for inshore ship detection. Due to the high similarity between the land and the ship body on gray level and texture features, the traditional methods are unable to achieve effective detection results [7].

In recent years, the level set method (LSM) has been widely applied in the fields of image processing [8] and computer vision. This method uses the geometric metrics of the curve such as the curvature and the normal vector to control the movement of the curve, so it does not depend on the parameters of the curve and can handle the changes of the topology. The level set method was first devised by Osher and Sethian [9] in 1988. Peng et al. [10] developed a Partial Differential Equation (PDE) based fast local level set method, and addressed two important issues that are intrinsic to the level set method. Adalsteinsson and Sethian [11] applied the Fast Marching Method, which is a very fast technique for solving the Eikonal and related equations, to the problem of building fast and appropriate extension velocities for the neighboring level sets. Chart and Vese [12] proposed a simplified Mumford-Sha model called the CV model in 2001. This model adds regularization terms in the energy function and is robust for images with Gaussian noise, but for images with intensity inhomogeneity, it often cannot perform a satisfactory segmentation result. Li et al. [13,14] proposed an effective Local Binary Fitting (LBF) model by introducing a Gaussian kernel function into the energy function. The model can effectively segment images with intensity inhomogeneity and avoids expensive reinitialization, but it is sensitive to initialization curve. Then Li et al. [15] proposed a distance regularized level set evolution (DRLSE) which eliminates the need for reinitialization and slightly avoids its induced numerical errors. Lv [16] integrated the fuzzy decision and a special local energy function to deal with vessel images. Wang [17] proposed an edge entropy fitting (EEF) energy, which is based on the LBF model, has achieved a reasonable segmentation result.

In the human visual system (HVS), it is the contrast not the brightness that occupies the most important part [18,19,20]. Itti [21] first proposed a saliency detection algorithm based on center-surround in 1998. The algorithm expresses three features (brightness, color, and direction) as a multi-scale Gaussian pyramid, and finally obtains a saliency map. In 2014, Chen et al. [22] proposed a local contrast measure (LCM) by measuring the difference between the current location and the local neighborhood, and then obtain a saliency map, which can achieve premier results. However, this algorithm will strengthen the false alarm point in the calculation process and increase the false alarm rate. Han et al. [23] proposed an improved LCM (ILCM) algorithm. The algorithm processed the image into sub-blocks and replaced the maximum value in the LCM algorithm with the mean value. Therefore, the false alarm rate is decreased. In recent years, visual saliency map has been applied to ship detection in SAR images [24]. In order to detect ship targets in SAR images, an optimal window selection mechanism based on the multiscale local contrast measure (LCM) is used in the local variance weighted information entropy (VWIE) [25].

The LBF is a well-known local information model which gets desirable segmentation results of images with intensity inhomogeneity. LCM and ILCM are two methods widely applied in computer vision. By calculating the saliency map, those two methods can improve the signal-to-noise ratio and get good performance on target detection.

Therefore, derived from LBF model and ILCM, an inshore ship detection method based on the level set method and visual saliency is proposed in this paper. Firstly, for the drawback that LBF model is sensitive to initialization curve, the image is quickly initialized by down-sampling. Then an improved level set method (improved LSM) based on the saliency map is proposed. The saliency map can improve the signal-to-noise ratio and solve the problem that LBF has poor segmentation results for SAR images. Finally, the adaptive threshold and discrimination are employed to obtain the final ship targets.

This paper is organized as follows. Related work and their detailed advantages or drawbacks are presented in Section 2. In Section 3, fast initialization, improved LSM, adaptive threshold and discrimination are described. Experimental results and analysis are given in Section 4. The conclusion is in Section 5.

## 2. Related Work

### 2.1. LBF Model

This paper mainly focuses on inshore ship detection, so we only consider a two-phase level set formulation. That is, the image is segmented into two disjoint regions, one is ship the targets region and the rest is the background region.

The LBF model [14] was proposed to deal with intensity inhomogeneity in the segmentation. The key steps are as follows.

The energy function $\varepsilon_{LBF}(\phi ,f_{1}(y),f_{2}(y))$ is defined as:

(1) $$\varepsilon_{LBF}(\phi ,f_{1}(y),f_{2}(y))=\sum_{i=1}^{2}\lambda_{i}\int K_{\sigma }(y-x)|I(x)-f_{i}(y){|}^{2}M_{i}(\phi (x))dx$$

where ϕ is the level set function, $f_{1}(y)$ and $f_{2}(y)$ are two values that approximate image intensities inside and outside ϕ=0. *H* is the Heaviside function, and $M_{1}(\phi )=H(\phi )$, $M_{2}(\phi )=1-H(\phi )$. $\lambda_{1}$ and $\lambda_{2}$ are weighting parameters. *K* is the Gaussian function with a standard deviation of σ, defined by:

(2) $$K_{\sigma }(y-x)=\exp(-{\left(y-x\right)}^{2}/2\sigma^{2})/\sqrt{2\pi }\sigma$$

after the iterations of energy function $\varepsilon_{LBF}(\phi ,f_{1}(y),f_{2}(y))$, image segmentation result is obtained by level set function ϕ.

**Figure 1:**
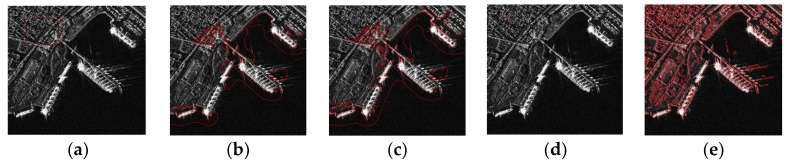
Results of LBF model with different initialization curves. (**a**) Initialization curve 1; (**b**) Result after 300 iterations with initialization curve 1; (**c**) Result after 3000 iterations with initialization curve 1; (**d**) Initialization curve 2; (**e**) Result after 300 iterations with initialization curve 2.

LBF model can effectively segment inhomogeneous images. However, it has two drawbacks:

1. It is sensitive to initial contour.
  Figure 1a,d are the original images with different initialization curves (red square). Figure 1b,c are the results after 300 iterations and 3000 iterations. No matter after how many iterations, level set function with initialization curve 1 cannot converge around the targets. Figure 1e is the result of Figure 1d after 300 iterations. It shows that the level set function with initialization curve 2 can well converge around the targets. By comparison, different initialization curves lead to different results. Therefore, it can be concluded that the LBF model is sensitive to initialization curve.
2. It has poor performances for SAR images segmentation.
  This is because the SAR images contain speckle noise, land architectures and have a low signal-to-noise ratio. Even for segmentation results with a proper initialization (as shown in Figure 1e), it still contains lots of clutters (including speckle noise and land architectures).

### 2.2. ILCM Method

In order to improve the signal-to-noise ratio of the targets, improved local contrast measure (ILCM) [23] is proposed based on the contrast mechanism. The key steps are as follows.

**Figure 2:**
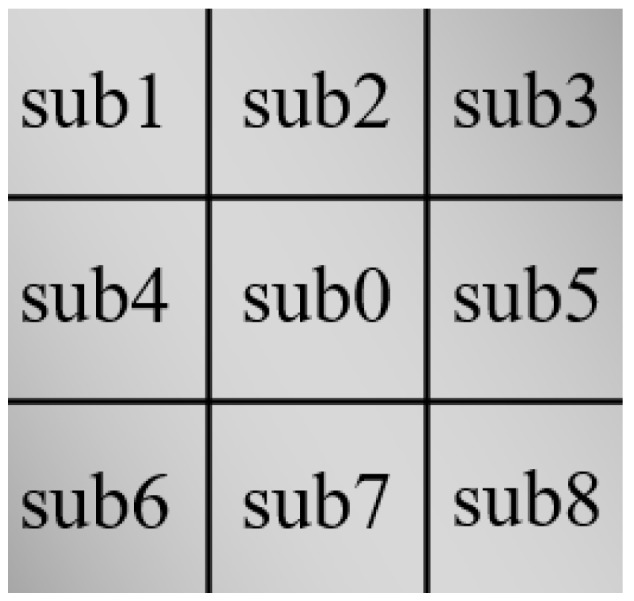
The locations of sub-blocks in block ϖ.

Let Ω be the image domain, for each point x∈Ω, its neighborhood called block ϖ. Divide ϖ into nine sub-blocks equally, marked as sub0, sub1, …, sub8. The locations of sub-blocks are shown in Figure 2.

The mean gray value of each sub-block sub(*i*) in the block ϖ is $m_{i}(i=0,1,\ldots 8)$, the maximum gray value in the middle sub-block sub0 is $L_{n}$, thus:

(3) $$m_{i}=\frac{1}{N}\sum_{j=1}^{N}I_{j}^{i}\;I_{j}^{i}\in \mathrm{sub}(i)$$

(4) $$L_{n}=\max(I_{j}^{0})\;I_{j}^{0}\in \mathrm{sub}0$$

The salient value of x∈Ω is defined as:

(5) $$I_{LCM}=\underset{i}{\min}\frac{L_{n}m_{0}}{m_{i}}\;i=1,2\ldots 8$$

for each x∈Ω, calculates its salient value according to (5), and then a saliency map is obtained.

The advantage of ILCM is that the targets in the saliency map have higher signal-to-noise ratio than in the original image.

The reason is as follows. For example, if sub0 is the target region, usually $\underset{i}{\max}m_{i}<L_{n}$, thus $I_{LCM}=\underset{i}{\min}\frac{L_{n}m_{0}}{m_{i}}=m_{0}\frac{L_{n}}{\underset{i}{\max}(m_{i})}>m_{0}$, then the target can be enhanced. In contrast, if sub0 is the patch around the target, usually $L_{n}<\underset{i}{\max}m_{i}$, thus $I_{LCM}=m_{0}\frac{L_{n}}{\underset{i}{\max}(m_{i})}<m_{0}$, then the pixel around the target can be suppressed.

Therefore, the salient value of the target pixel is larger than its original grayscale, and the salient value of the pixel around the target is smaller than its original grayscale, thus the signal-to-noise ratio of target in the saliency map is enhanced. That is to say, the targets become more salient.

## 3. Proposed Method

Considering the advantages and drawbacks of LBF model and ILCM method above, an inshore ship detection based on the level set method and visual saliency is proposed.

**Figure 3:**
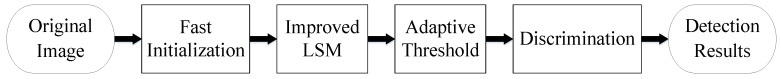
Flowchart of the proposed method.

Firstly, because the LBF model is sensitive to the initialization curve, the image is quickly initialized by down-sampling. Secondly, improved LSM is proposed to get level set function. The improved LSM, to be specific, is a level set method improved by visual saliency. Thirdly, candidate targets are gained by adaptive threshold of level set function. Finally, discrimination is employed to select final ship targets. The specific flowchart is shown in Figure 3.

### 3.1. Fast Initialization

**Figure 4:**
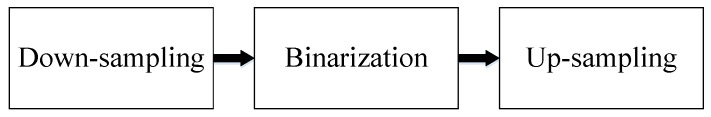
Flowchart of the proposed fast initialization.

This paper focuses on inshore ship detection. Inshore ships are usually large targets with certain sizes. Inspired by the objectless Binarized Normed Gradients (BING) algorithm [26], a fast initialization procedure is applied to our proposed method. The flowchart of the fast initialization is shown in Figure 4.

First, down-sampling the original image, that is, rescale the image into a smaller size. However, the rescale size must be smaller than the minimum size of the ship targets. Only in this way, even in the down-sampled image, there still have target pixels.

Then, binarize the down-sample image. The gradient of the down-sample image changes little, so it is easier to do the segmentation in the down-sample image. The threshold of binarization is obtained through the traditional Otsu [27] algorithm. After binarization, the image is segmented into the target part and background part.

Finally, up sample the binary image and the final initialization curve is obtained. Of course, this curve is relatively coarse, but it is enough to be used as an initialization curve.

### 3.2. Improved LSM

Improved LSM is short for the improved level set method, which specifically refers to a level set method based on visual saliency fitting energy.

First, the saliency map is calculated by the improved local contrast measure (ILCM).

For each point x∈Ω in the image, the calculation equation of the saliency map is rewritten as follows:

(6) $$S(x)=ILCM=\underset{i}{\min}\frac{L_{n}m_{0}}{m_{i}}$$

Second, the calculation of improved LSM is mainly based on the saliency map, because the targets are more salient and the signal-to-noise ratio is higher in the saliency map.

Let $\Omega_{1}$ and $\Omega_{2}$ be the target region and background region respectively, and they satisfy $\Omega_{1}\cup \Omega_{2}=\Omega$ and $\Omega_{1}\cap \Omega_{2}=\emptyset$, then we consider a neighborhood with a radius at each point y∈Ω, defined as $O_{y}≜\left\{x:|x-y|\leq \rho \right\}$.

According to the LBF model, we define a visual saliency fitting energy $\varepsilon (\phi ,s_{1,}s_{2})$ as:

(7) $$\varepsilon (\phi ,s_{1,}s_{2})=\sum_{i=1}^{2}{\iint K_{\sigma }(x,y)|S(x)-s_{i}(y)|}^{2}H_{i}(\phi (x))dxdy$$

where ϕ is the level set function, S(x) is the salient value in the neighborhood of the position *y*. $s_{1}(y)$ and $s_{2}(y)$ are the mean value of the saliency intensity of $O_{y}\cap \Omega_{1}$ and $O_{y}\cap \Omega_{2}$ respectively.

$\varepsilon (\phi ,s_{1},s_{2})$ denotes the data term. In order to regularize the level set function ϕ, two widely used regularization terms L(ϕ)=∫|∇H(ϕ)|dx and $R_{p}(\phi )=\int p(|\nabla \phi |)dx$ are adopted in our energy function. L(ϕ) computes the arc length of the level contour of ϕ=0, $R_{p}(\phi )$ is the distance regularized level set formulation [28], the function *p* is defined as $p(s)=(1/2){\left(s-1\right)}^{2}$.

Thus, the final energy function is given by:

(8) $$F(\phi ,s_{1},s_{2})=\varepsilon (\phi ,s_{1},s_{2})+\nu L(\phi )+\mu R_{p}(\phi )$$

where ν and μ are the weighting parameters.

The final energy $F(\phi ,s_{1},s_{2})$ is minimized by using the standard gradient descent method. With minimized $F(\phi ,s_{1},s_{2})$, we can obtain the result of image segmentation given by the level set function ϕ.

Keeping $s_{1}(y)$ and $s_{2}(y)$ fixed, the evolution formula of the level set function is:

(9) $$\frac{\partial \phi }{\partial t}=-\delta (t)(e_{1}-e_{2})+\upsilon \delta (\phi )div(\frac{\nabla \phi }{\left|\nabla \phi \right|})+\mu div(d_{p}(|\nabla \phi |)\nabla \phi )$$

where ∇ is the gradient operator, div(.) is the divergence operator, and the $d_{p}$ and $e_{i}$ in (9) are defined as:

(10) $$d_{p}(s)≜\frac{p^{\prime }(s)}{s}$$

(11) $$e_{i}(y)=\int K_{\sigma }(x,y)|S(x)-s_{i}(y){|}^{2}dy$$

Meanwhile, for a fixed level set function ϕ, we minimize the function $F(\phi ,s_{1},s_{2})$ with respect to the functions $s_{1}(y)$ and $s_{2}(y)$ which satisfy the following Euler–Lagrange equations:

(12) $$\int K_{\sigma }(x,y)(S(x)-s_{i}(y))H_{i}(\phi (x))dx=0\;i=1,2$$

Then we obtain:

(13) $$s_{i}(y)=\frac{\int K_{\sigma }(x,y)S(x)H_{i}(\phi (x))dx}{\int K_{\sigma }(x,y)H_{i}(\phi (x))dx}$$

Through all those equations, with minimized final energy function $F(\phi ,s_{1},s_{2})$, we can obtain the result of image segmentation given by the level set function ϕ.

### 3.3. Adaptive Threshold

The level set method is usually applied to magnetic resonance (MR) image segmentation. MR images are segmented into two parts by the curve of the level set function (ϕ=0). However, for SAR images, segmentation is more complex because of speckle noise as well as land architectures. Therefore, in order to detect targets better from the background, an adaptive threshold is proposed.

Inspired by Constant False-Alarm Rate (CFAR) detection algorithm [29], we define the adaptive threshold *T* as:

(14) $$\sum_{T-1}^{+\infty }\frac{h_{i}(s)}{n}<p\leq \sum_{T}^{+\infty }\frac{h_{i}(s)}{n}$$

where $h_{i}(s)$ is the frequency of gray value *s*, n is the total number of pixels of level set function ϕ. *p* is approximately the proportion of targets in the entire image, and it is an empirical value. The value of *p* is different for different type of images. And for ship detection result, it is more important not to lose targets than to have some clutters, so *p* could set to be a little larger.

### 3.4. Discrimination

After the procedure of adaptive threshold, ship targets can be well detected. However, it is still possible that some clutter with similar intensity to targets cannot be removed, so further discrimination is needed.

Because the detection result of proposed method has good connectivity and integrity (detailed explanation is in Section 4.3), and most of the strong clutters remained in detection result is spot-like or small-area blocks, the targets can be detected with a simple discrimination based on area size. Specific steps are as follows:

1. Traverse the entire result image, search all the closed regions and number them $R_{i}$, where 0 ≤ i ≤ N, N denotes the number of closed regions.
2. Calculate the area size of each region $A_{i}$.
3. Set two thresholds of area size according to the image type, the smaller one named $T_{l}$, and the larger one named $T_{u}$.
4. If $A_{i}$ ≤ $T_{l}$ or $A_{i}$ ≥ $T_{u}$, $R_{i}$ is determined as a false alarm target and is removed from the detection result. Otherwise, it can be a target and then move to the next region.
5. If all of the regions are tested, we can obtain the final detection result.

## 4. Experiment Results and Discussion

**Figure 5:**
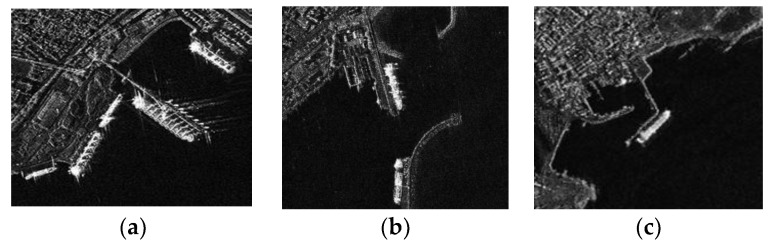
Experimental SAR images. (**a**) Image 1; (**b**) Image 2; (**c**) Image 3.

To evaluate our proposed algorithm quantitatively, we implemented experiments to three SAR images of inshore ships as shown in Figure 5. Figure 5a–c shows the images of Visakhapatnam port and its nearby waters in India acquired by the TerraSAR-X satellite in 2008. The detailed parameters (including the polarization, band, size, and resolution) of the three images are listed in Table 1. These three images are corrupted by speckle noise to different degrees, and contain a large number of land clutters. The performance of the algorithm was tested using MATLAB software on a computer equipped with a 2.8 GHz Intel i7 processor and 8.0 GB memory.

### 4.1. Results and Analysis of Fast Initialization

**Figure 6:**
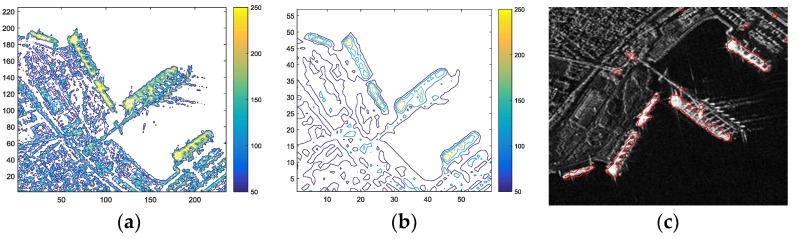
Results of fast initialization. (**a**) Contour map of the original image; (**b**) Contour map of the down-sample image; (**c**) Result of initialization curve in the original image.

To reveal the advantages of the fast initialization, we rescale the image into one-quarter of the original image, and then we can obtain:

As revealed in Figure 6a, the original image contains lots of clutters (including speckle noise and land architectures).

Figure 6b is the contour map of the down-sample image. It has less clutters than Figure 6a, which is more beneficial for image segmentation. The red curve in Figure 6c is the initialization curve after image binarization and up sampling. Although it is a coarse curve compared with the true edge of ship targets and contains some clutters area, the ship targets have been roughly outlined.

This procedure has two advantages:

1. Down-sampling accelerates the speed of computation. Because the number of pixels in the image is greatly reduced.
2. Down-sampling can remove the influence of isolated clutter points. For example, rescale the image into one-quarter of the original image, noise whose size is less than one pixel will not appear in the rescaled image.

All those advantages result in getting a faster and better initialization curve, and a better initialization curve is beneficial to the evolution of the proposed LSM.

### 4.2. Results and Analysis of Proposed LSM

The proposed LSM is improved on the basis of the LBF model. In order to verify the validation of the improvement, the comparison between LBF model and the proposed LSM is analyzed in this section.

When calculating the saliency map, the size of ϖ takes 9×9 and the middle block sub0 takes 3×3. In the level set evolution, the Gaussian kernel function σ takes 4, μ takes 1 and ν takes $0.001\times 255^{2}$, the time step Δt takes 0.1.

In the calculation, the Heaviside function *H* is replaced by a smooth function $H_{\varepsilon }$, which is defined by:

(15) $$H_{\varepsilon }(x)=\frac{1}{2}\left[1+\frac{2}{\pi }\arctan(\frac{x}{\varepsilon })\right]$$

where ε=1, the Dirac delta function δ is replaced by $\delta_{\varepsilon }$, which is defined by:

(16) $$\delta_{\varepsilon }(x)=H_{\varepsilon }^{\prime }(x)=\frac{1}{\pi }\frac{\varepsilon }{\varepsilon^{2}+x^{2}}$$

To avoid the effects of initialization, the same initialization curve obtained in section 4.1 is used for these two methods. Through the calculation of LBF model and the proposed LSM, we can get level set function.

**Figure 7:**
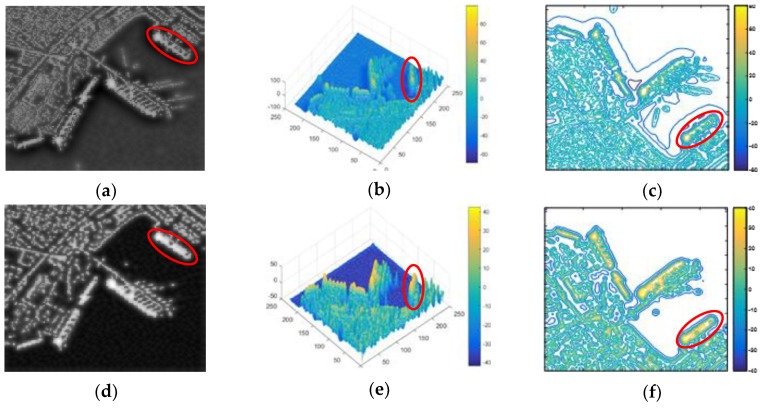
Results of LBF model (**a**–**c**): (**a**) The level set function; (**b**) The mesh map of level set function; (**c**) The contour map of level set function. Results of the proposed LSM (**d**–**f**): (**d**) The level set function; (**e**) The mesh map of level set function; (**f**) The contour map of level set function.

Level set function after LBF model and the proposed LSM use the same initialization curve, so the only difference is the improvement based on visual saliency. From Figure 7a,d, it can be seen that the targets in the result of proposed LSM are more salient. To display the results better, the mesh map and contour map of Figure 7a,d are shown in Figure 7b,c and 7e,f respectively. By comparing the Figure 7b,e or Figure 7c,f, we can conclude that the targets are more salient in the result of the proposed LSM (take the target in the red circus as an example). That is to say, the contrast between targets and background is larger after the improvement based on visual saliency.

The targets in the saliency map have higher signal-to-noise ratio than in the original image. The calculation of the proposed LSM is mainly based on the saliency map. Hence, the targets are more salient in the result of the proposed LSM.

### 4.3. Results and Analysis of Adaptive Threshold

MR images are segmented into two parts by level contour of ϕ=0. However, in this paper, adaptive threshold is used for ship detection. Therefore, zero (ϕ=0) and the adaptive threshold of the level set function are compared in this section.

In this article, all images are the same type, they are all inshore ship images with large ship targets. For our experimental images, the proportion of targets in the entire image ranges from 0.005 to 0.035, so *p* takes 0.06 for all images.

For image 1, though (14), *p* takes 0.06, then we can set the adaptive threshold *T* to 10. After the adaptive threshold of the level set function, candidate targets are obtained.

**Figure 8:**
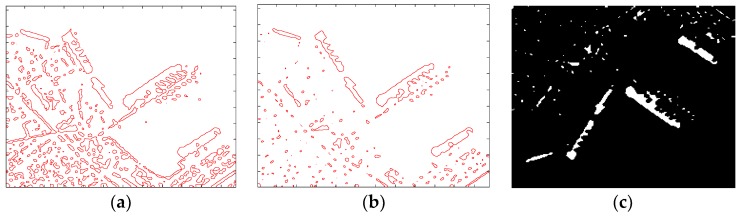
Result after adaptive threshold. (**a**) Zero level set function (*T* = 0); (**b**) Adaptive threshold for level set function (*T* = 10 for image 1); (**c**) Detection result after adaptive threshold.

It can be visually observed that the result of the adaptive threshold (Figure 8b) has less clutters than zero level set function (Figure 8a). This is because the contrast between targets and background is larger after the improvement based on visual saliency, and appropriately raise the threshold can remove most clutters.

Moreover, the results of ship targets have good connectivity and integrity (Figure 8c). The reason for this property is that:

1. The level set method is an active contour model which aim to identify each region of interest by using a certain region descriptor to guide the motion of the active contour [26]. It is different from the method (like CFAR) which needs a pixel-by-pixel comparison, the result of it is consist of regions, so the level set method is more like a region detector.
2. The characteristics of the saliency map. For isolated dark spots in the ship targets, a higher saliency value can be obtained by the ILCM algorithm. Because in the formula (4), $L_{n}$ is the maximum value in the sub0 region, as long as there is one point with a high gray value in the sub0 region, then the salient value of dark spot become larger. In this way, the isolated dark spot inner the ship target also has a higher intensity in the saliency map.

### 4.4. Results and Analysis of Discrimination

Although the detection result in Figure 8c has preferable performance, it still contains some clutters with similar intensity to targets. Further discrimination based on the area size is required to get accurate ship targets.

**Figure 9:**
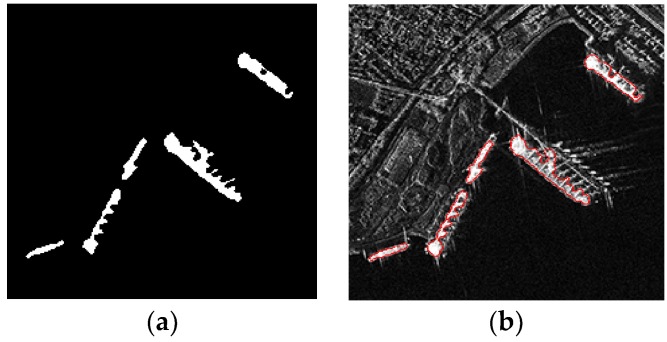
Results after discrimination (with threshold parameters $T_{l}$ sets to 100, and $T_{u}$ undesired). (**a**) Final detection result after discrimination; (**b**) Final detection result curve in original image.

Final detection results after discrimination and the final result curve in the original image are shown in Figure 9a,b, respectively. It can be seen all the ship targets have been detected, and the final detection result is accurate, integrity and almost has no clutter points. The detailed analysis of the result is described in the next section.

### 4.5. Results and Analysis of the Whole Proposed Method

#### 4.5.1. Comparison with Other Two Ship Detection Methods

In order to validate the effectiveness of the proposed method, it is compared with *K*-CFAR and the Multiscale Variance Weighted Image Entropy (MVWIE) method [25]. *K*-CFAR is the traditional method of ship detection. *K* distribution is one of the most widely used models for statistical modeling of SAR images [29]. MVWIE is a state-of-the-art ship detection method based on multiscale local contrast measure (multiscale LCM) and information entropy, which can effectively detect ship targets from the complex background SAR images.

Firstly, the proposed method before discrimination is compared with *K*-CFAR and MVWIE. Then same discrimination described in Section 3.4 is employed to the results of these three methods.

**Figure 10:**
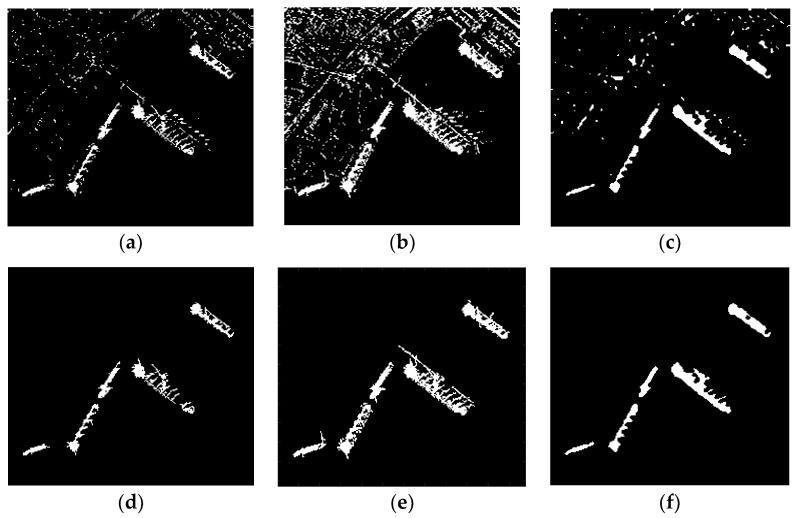
Detection results of three methods for image 1 (**a**–**c**): (**a**) *K*-CFAR; (**b**) MVWIE; (**c**) The proposed method. Detection results of three methods for image 1 after discrimination (**d**–**f**, with threshold parameters $T_{l}$ sets to 100, and $T_{u}$ undesired): (**d**) *K*-CFAR; (**e**) MVWIE; (**f**) The proposed method.

Figure 10a, d are detection results of *K*-CFAR before and after discrimination. It can be seen that the detection result of *K*-CFAR is poor and some target pixels is missed. *K*-CFAR is more suitable for homogeneous background. Inshore ship images have many edges and architectures, so its performance is not preferable.

MVWIE performs well in detecting ships, but it also remains much clutters in the result (Figure 10b). Although most of the clutter can be removed after discrimination, there are still some clutters connected to the ship remained (Figure 10e). These clutters could change the shape of the ship in the final result, which results in an incorrect detection result.

The proposed method recognizes all the ship targets and there are some clutters in the detection result (Figure 10c). In contrast with *K*-CFAR and MVWIE, the final detection result of the proposed method has good connectivity and integrity for entire ship targets, and it almost has no land clutters connected with ships (Figure 10f).

#### 4.5.2. Quantitative Evaluation

Additionally, in order to evaluate the proposed method quantitatively, the experimental results of these three methods are compared.

First, the detection rate ($P_{D}$) and the false detection rate ($P_{F}$) are used to evaluate the performance quantitatively [7]. Where:

(17) $$P_{D}=\frac{T_{p}}{S_{p}}\times 100\%$$

$T_{p}$ is the number of detected pixels belonging to the ship, and $S_{p}$ is the number of all these ship pixels. And:

(18) $$P_{F}=\frac{F_{p}}{D_{p}}\times 100\%$$

$F_{p}$ denotes the number of false alarm pixels, and $D_{p}$ is the number of all these detected pixels.

Without loss of generality, all the detection rate and false detection rate of the three methods on image 1–3 in Table 2 are derived under the same discrimination procedure (described in Section 3.4).

**Figure 11:**
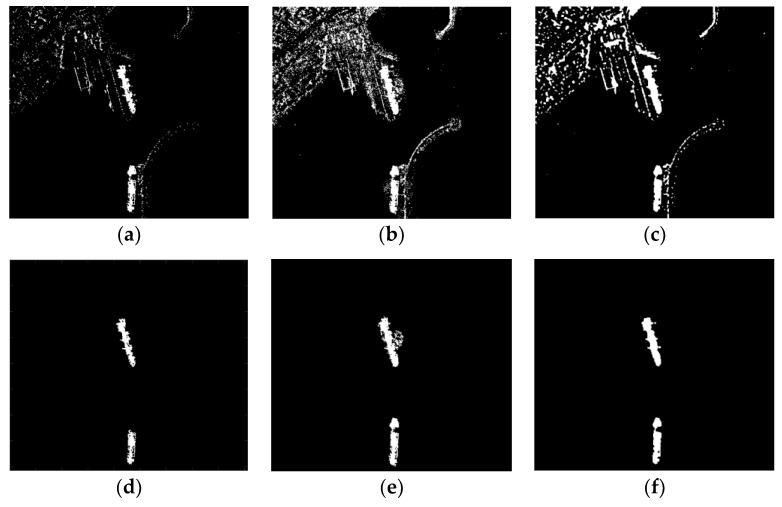
Detection results of three methods for image 2 (**a**–**c**): (**a**) K-CFAR; (**b**) MVWIE; (**c**) The proposed method. Detection results of three methods for image 2 after discrimination (**d**–**f**, with threshold parameters $T_{l}$ sets to 500, and $T_{u}$ undesired): (**d**) K-CFAR; (**e**) MVWIE; (**f**) The proposed method.

**Figure 12:**
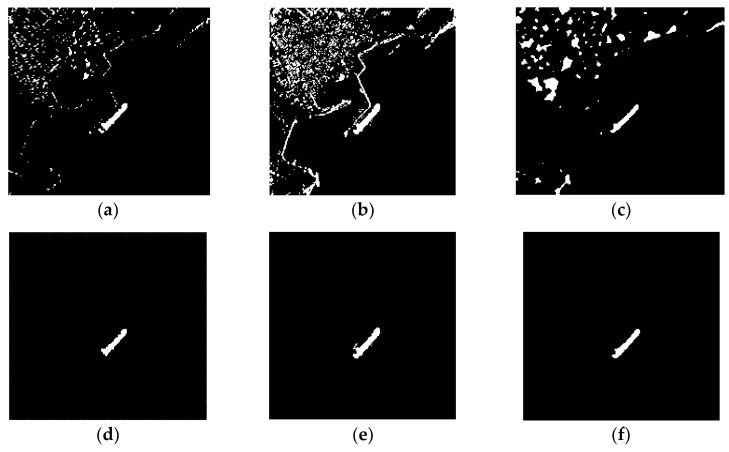
Detection results of three methods for image 3 (**a**–**c**): (**a**) K-CFAR; (**b**) MVWIE; (**c**) The proposed method. Detection results of three methods for image 3 after discrimination (**d**–**f**, with threshold parameters $T_{l}$ sets to 200, and $T_{u}$ undesired): (**d**) K-CFAR; (**e**) MVWIE; (**f**) The proposed method.

Table 2 shows the detection rate and false detection rate of these three methods based on image 1 (Figure 10d–f), image 2 (Figure 11d–f) and image 3 (Figure 12d–f).

As shown in Table 2, the results of *K*-CFAR have lower $P_{F}$, but the $P_{D}$ is also lower. The results of MVWIE have higher $P_{D}$, but the $P_{F}$ is also higher. However, the results of the proposed method have higher $P_{D}$ and lower $P_{F}$ at the same time.

Then, the efficiencies of the algorithms were compared. Table 3 reports the total time cost (include the discrimination procedure) for different methods.

As we can see in Table 3, for the same method, computational time is related to the sizes of images. In MVWIE method, multiscale LCM provides an ideal selection mechanism for the local optimal windows, the size of some windows could be a little large, so its computational time is longer than the K-CFAR method. Our proposed method uses ILCM with a fixed small window, but the iteration of level set method costs some time, so the computational time is little longer than the MVWIE method. However, the performance of the proposed method is much better than other two methods.

#### 4.5.3. Generalization Ability of the Proposed Method

**Figure 13:**
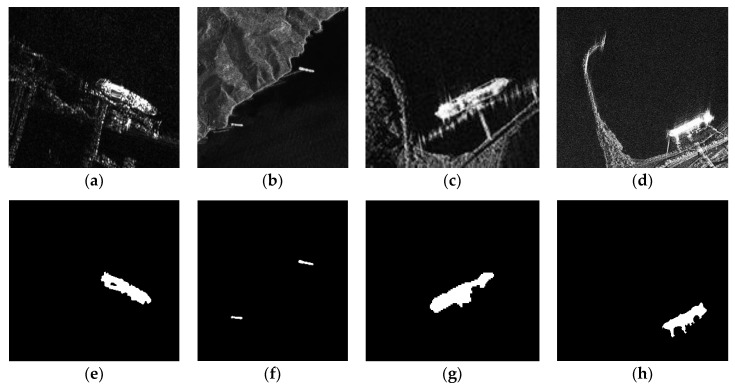
Generalization ability of the proposed method. The original images (**a**–**d**): (**a**) Image 4; (**b**) Image 5; (**c**) Image 6; (**d**) Image 7. The detection results (**e**–**h**): (e) Image 4 (with threshold parameters $T_{l}$ sets to 100, and $T_{u}$ undesired); (**f**) Image 5 (with threshold parameters $T_{l}$ sets to 100, and $T_{u}$ sets to 200); (**g**) Image 6 (with threshold parameters $T_{l}$ sets to 200, and $T_{u}$ undesired); (**h**) Image 7 (with threshold parameters $T_{l}$ sets to 1700, and $T_{u}$ undesired).

To further prove the generalization ability of the proposed method, we employed the proposed method to four more TerraSAR-X images as shown in Figure 13. Figure 13a–d show the images acquired by the TerraSAR-X satellite under different scenarios. The detailed parameters (including the polarization, band, size, resolution, acquisition location and acquisition year) of the SAR images are shown in Table 4.

The size and amount of land clutters are different in images of Figure 13, and these images are corrupted by speckle noise to different degrees due to their different acquisition conditions. The experiment results illustrate that our method also has effective performance on these images, and prove the generalization ability of the proposed method.

## 5. Conclusion

In this paper, an inshore ship detection method based on the level set and visual saliency is proposed. Firstly, to overcome the drawback of the LBF model, a fast initialization method is employed. Through down-sampling, the initialization method can speed up the computation and removes the influence of isolated clutters, which results in a better initialization curve. Secondly, an improved LSM based on visual saliency is proposed, the saliency map has a higher signal-to-noise ratio and the local level set method can effectively segment images with intensity inhomogeneity. Thirdly, the adaptive threshold rather than zero is used to obtain potential targets. Finally, ship targets are selected through the area discrimination. Experimental results show that the proposed method is robust to speckle noise, land clutters in SAR images and can detect ship targets with accuracy and integrity. Compared with *K*-CFAR and MVWIE, the proposed method has a higher detection rate and a lower false alarm rate. Experiments on a number of inshore ship images prove the generalization ability of the proposed method.

However, this paper focuses on the inshore ship with large sizes, the detection for tiny ship targets will be considered in the following work.

## Figures and Tables

**Table 1:** Parameters of the SAR images in Figure 5, where HH and VV denote the polarization models for horizontal transmit and horizontal receive, and vertical transmit and vertical receive, respectively.

Image Name	Polarization	Band	Size (pixels)	Resolution
Image 1	HH	X	235 × 225	1.0 m × 1.0 m
Image 2	HH	X	456 × 407	1.0 m × 1.0 m
Image 3	VV	X	200 × 200	3.0 m × 3.0 m

**Table 2:** Detection rate and false detection rate of these three methods. ($P_{D}$/$P_{F}$).

Image	*K*-CFAR	MVWIE	Proposed Method
image 1	63.2/12.0 ^1^	80.5/32.3	89.5/4.7
image 2	69.2/4.5	91.1/21.2	93.5/1.9
image 3	82.6/3.9	95.7/17.8	96.2/1.3

^1^ Detection rate /false detection rate.

**Table 3:** Time (in seconds) cost of the three algorithms on image 1–3.

Image	*K*-CFAR	MVWIE	Proposed Method
image 1	6.00	9.14	11.46
image 2	10.44	41.72	45.61
image 3	4.68	9.18	9.53

**Table 4:** Parameters of the SAR images in Figure 13, where HH and VV denote the polarization models for horizontal transmit and horizontal receive, and vertical transmit and vertical receive.

Images	Polarization	Band	Size (pixels)	Resolution	Location	Year
Image 4	HH	X	364 × 244	3.0 m × 3.0 m	Shanghai, China	2010
Image 5	VV	X	357 × 381	3.0 m × 3.0 m	Kochi, India	2008
Image 6	HH	X	175 × 116	1.0 m × 1.0 m	Visakhapatnam, India	2008
Image 7	VV	X	331 × 292	1.0 m × 1.0 m	Kerch, Russia	2009
